# Carriers of *COL3A1* pathogenic variants in Denmark: Interfamilial variability in severity and outcome of elective surgical procedures

**DOI:** 10.1111/cge.14176

**Published:** 2022-07-04

**Authors:** Sofus Sølyst, Riina Oksjoki, Stense Farholt, Dorte Guldbrand Nielsen, Alex H. Christensen, Christina R. Fagerberg, Lotte Risom, Pernille Axél Gregersen, Maria Bejerholm Christensen, Torsten Bloch Rasmussen, Birgitte Rode Diness

**Affiliations:** ^1^ Department of Clinical Genetics Copenhagen University Hospital Rigshospitalet Copenhagen Denmark; ^2^ Department of Clinical Medicine, Faculty of Health and Medical Sciences University of Copenhagen Copenhagen Denmark; ^3^ Department of Cardiology Copenhagen University Hospital, Herlev‐Gentofte and Rigshospitalet Copenhagen Denmark; ^4^ Department of Cardiology Aarhus University Hospital Aarhus Denmark; ^5^ Center for Rare Diseases, Pediatric and Adolescent Medicine Copenhagen University Hospital Rigshospitalet Copenhagen Denmark; ^6^ Center for Rare Diseases, Pediatric and Adolescent Medicine Aarhus University Hospital Aarhus Denmark; ^7^ Department of Clinical Genetics Aarhus University Hospital Aarhus Denmark; ^8^ Department of Clinical Genetics Odense University Hospital Odense Denmark

**Keywords:** *COL3A1*, genotype–phenotype, surgical complications, vascular EDS, vascular Ehlers–Danlos syndrome, vEDS

## Abstract

The study describes all patients in Denmark with vascular Ehlers–Danlos syndrome (vEDS). Carriers of pathogenic or likely pathogenic *COL3A1* variants were retrospectively identified through registries and specialized clinics. Medical records were reviewed for vascular‐ or organ ruptures and invasive procedures performed. Identified families were divided by variant type (null, splice, and missense) and familial phenotypes (severe or attenuated). Families in which at least one carrier has suffered a major event before the age of 30 were classified as severe, whereas families in which at least three carriers had reached the age of 40 without a major event were classified as attenuated. Eighty‐seven persons (59 still alive) from 25 families were included with a mean observation time of 44 years. Sixty‐seven percent of patients could be subclassified in a familial phenotype. Thirty‐one major events were observed. Eleven complications in 172 invasive procedures were recorded. No fatal complications to elective surgery were observed. The type of *COL3A1* variant did not reliably predict phenotype, but a pattern of intrafamilial consistency emerged with some families showing an attenuated form of vEDS. Elective medical procedures appear to be safer than previously thought, although data only allow for conclusions regarding individuals from families with the attenuated form of vEDS.

## Graphical abstract

We report on all known Danish cases of Vascular Ehlers–Danlos Syndrome (87 individuals from 25 families). Main findings:

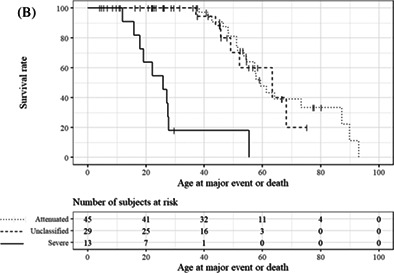

- Some families have a severe phenotype (at least one carrier had a major event before the age of 30 years), whereas other families seem to have an attenuated phenotype (at least three carriers reached the age of 40 years without a major event). The type of *COL3A1* variant did not reliably predict severity
- No fatal complications to 125 elective medical procedures were observed

## 1 INTRODUCTION

Vascular Ehlers–Danlos Syndrome (vEDS) is one of 13 different types of Ehlers–Danlos Syndrome.^1^ The phenotype of vEDS includes an increased risk of vascular complications such as aneurysms, dissections, and ruptures of large‐ and medium‐sized arteries, as well as hollow organ rupture.^2^, ^3^ This leads to a significant reduction in life expectancy with a reported median age at death of 51 years.^4^

vEDS is an autosomal dominant condition caused by pathogenic variants in *COL3A1*.^3^ *COL3A1* is translated into type III pre‐procollagen, which undergoes co‐ and post‐translational modifications allowing three type III procollagen molecules to assemble into the triple‐helical type III collagen.^5^ In the extracellular matrix of connective tissue, cross‐links are made between these molecules, creating macromolecular fibrils which, in turn, combine into fibers.^6^ These type III collagen fibers provide a tissue with tensile strength and are abundant in tissues such as blood vessels and hollow organs.^7^ Pathogenic variants in *COL3A1* lead to either a reduced amount or an abnormal structure of type III collagen, resulting in varying degrees of connective tissue fragility.^8^

Variants in *COL3A1* presumed to cause haploinsufficiency have previously been shown to result in a milder phenotype than missense or splice variants.^9^, ^10^, ^11^ The triple‐helical nature of collagen is assumed to be the biological background for this phenomenon, as haploinsufficiency results in a quantitative defect, whereas variants resulting in structural changes lead to a qualitative defect of type III collagen with abnormal triple‐helix folding. As three procollagen molecules combine in one triple‐helical collagen III molecule, in theory, only one in eight of these molecules will be formed from three normal procollagen molecules assuming equal translation from the two alleles. The abnormal folding of the triple helices results in a dominant‐negative effect with an 87% reduction in the production of structurally normal type III collagen, whereas haploinsufficiency theoretically only reduces the amount by 50%.^12^, ^13^

Optimal surveillance and management of patients with vEDS is a challenge. The fragility of connective tissues, hollow organs, and arteries has led to recommendations for extreme caution regarding pregnancy, surgery, and minor invasive diagnostic procedures because of the presumed high risk of complications.^3^, ^14^, ^15^, ^16^ However, the studies on which guidelines are based have a high proportion of index patients and patients diagnosed following an event.^16^, ^17^ They may, therefore, be less predictive in the genomic era where the diagnosis is more often made prior to events, as part of cascade screening or as an incidental finding. This retrospective Danish national multicenter study aims to contribute to the understanding of the variable expressivity by including observational data for all known patients including those diagnosed by familial workup and by examining whether a subclassification based on familial phenotype could create the empirical basis for changed management of *COL3A1* variant carriers from less severely affected families.

## 2 SUBJECTS AND METHODS

### 2.1 Study population

Index patients with pathogenic or likely pathogenic variants in *COL3A1* were identified through the six Departments of Clinical Genetics, the two national Centers for Rare Diseases in Denmark, and through specialized units for inherited cardiac conditions. Relatives to index patients with the familial *COL3A1* variant were identified similarly, through additional cascade screening, and by pedigree analysis. Obligate carriers were also included. Identified patients were invited to the study and included upon written informed consent. For deceased patients, consent was provided by living relatives. The genetic status of a deceased individual at risk of carrying a familial variant was sought determined by genetic analysis of saved specimens from surgical procedures or Guthrie cards. We did not include individuals of unknown genetic status as cases even if they had an event that could potentially be due to vEDS.

### 2.2 Genetic analysis

Results of genetic analyses were available from standard clinical investigations with Sanger or Next‐Generation Sequencing of *COL3A1* alone, in a multi‐gene panel, or through whole exome‐ or genome sequencing and in some instances supplementary sequencing of mRNA from cultured fibroblasts. For deceased individuals identified as at risk through the study, DNA from saved specimens was analyzed when available.

### 2.3 Analysis of effect and segregation of variants

The effect of the variant was analyzed using Alamut® Visual Software v. 2.15. Variants were classified according to ACMG guidelines^18^ using the VarSome database.^19^ Only patients with pathogenic or likely pathogenic variants were included in this study. Variants were divided into three groups: missense, null variants, and splice site variants. Splicing prediction was performed using the MaxEntScan prediction program.^20^ Splice site variants of *COL3A1* may result in functional null variants or in‐frame exon skipping and full translation of a shortened protein product and were therefore treated as a separate category.^9^, ^10^

### 2.4 Data sources

Data were collected retrospectively from clinical records, death certificates, pathology reports, and genetic test reports: date of birth, *COL3A1* variant, age at ascertainment, minor and major clinical vEDS features,^17^ medical treatment, major events, and cause and date of death. For recorded procedures, date, indication, specific procedure, and complications were registered. Data from birth to death or January 1, 2021 were included, and time from birth to death or January 1, 2021 was considered observation time. Time from diagnosis to death or January 1, 2021 was considered follow‐up time.

In Denmark, all inhabitants have a unique social security number from birth to death, and almost, all health care and diagnosis are delivered through the publicly financed national health care system and tracked in national registries based on this number. Thus, Danish registries permit complete data regarding hospitalizations, outpatient procedures, and histological examinations.^21^ This allows for a very high degree of retrospective data completeness for both index and relatives following diagnosis. The date and cause of death are registered as well. For study subjects who died prior to the establishment of these registries, death certificates were inspected. Information regarding surgery and invasive diagnostic procedures was enriched with medical histories reported by patients or relatives whenever possible.

Pregnancies and births were recorded from pedigrees and population registries, and complications in pregnancy and delivery as well as mode of delivery were recorded from clinical records and patient reports when possible.

### 2.5 Definitions

Major events were defined as rupture or dissection of large‐ or medium‐sized arteries or organ rupture. Colon perforations caused by diverticular disease were not regarded as major events as the current International Classification of the Ehlers–Danlos Syndromes specifically excludes this from the major diagnostic criteria of vEDS.^1^

Age of ascertainment was determined using the date the diagnosis was confirmed by genetic testing, or in case of post‐mortem diagnoses, the date of the first major event or date of death.

Surgery and invasive diagnostic procedures were defined as any instance of instruments larger than a syringe either piercing the skin, mucous membranes, or the tympanic membrane, passing the oropharynx, or entering any other body orifice.

Complications to surgery and invasive diagnostic procedures were defined as any undesirable, unintended, and direct result of the procedure affecting the patient, which would not have occurred had the procedure gone as well as could reasonably be hoped.^22^

### 2.6 Categorization of families

Families with pathogenic/likely pathogenic *COL3A1* variants were subclassified according to the severity of their phenotype in severe or attenuated familial phenotype:

- Severe familial phenotype: Families in which one or more *COL3A1* variant carriers suffered a major event before the age of 30 years.
- Attenuated familial phenotype: Families in which no individual suffered a major event before the age of 30 years and more than three carriers of the *COL3A1* variant carriers had lived past the age of 40 without a major event and no more than one‐third of all known carriers had suffered a major event between 30 and 40, counting only carriers with major events or those having reached the age of 40 without one.

The categorization was based on clinical observations aiming for a balance between allowing categorization as early as possible while respecting the limitations in information available in small families and de novo cases.

Families that did not fulfill the criteria for either severe or attenuated phenotypes were considered unclassified.

### 2.7 Statistical analysis

All statistical analyses were performed with the R Studio software version 1.3.1093. *p* < 0.05 was considered significant. Categorical variables were analyzed with a chi‐square test or Fisher's exact test, when appropriate. Plots were created using Kaplan–Meier analysis showing the time to the first major event or the subject's death. All living subjects were censored on the last known date they were alive without having experienced a major event. The patients were grouped by familial phenotype as well as variant type. Age at event or death was compared between groups using a log‐rank test.

## 3 RESULTS

### 3.1 Enrollment

A total of 87 carriers of pathogenic or likely pathogenic *COL3A1* variants including 25 index patients were identified. Fifty‐nine of 88 *COL3A1* carriers were alive and available for clinical phenotyping. The mean observation time was 44 years. Follow‐up time (diagnosis to death/end of observation) was 0 for 24 patients. The mean follow‐up time for the remaining 63 patients was 3.8 years (range 0.04–18). All patients were encouraged to have a full vascular evaluation following diagnosis and to attend regular surveillance. Medical histories were available for 78 of the subjects, and for nine deceased patients, only census data and death certificates were available. No patients declined participation. For 15 of the enrolled families, good pedigree information was available. In five of these families, a total of nine deceased relatives were reported to possibly have had symptoms compatible with vEDS, but genotype could not be determined and they were not included in the study.

### 3.2 Subclassification

Each participant was assigned to a familial phenotype and a variant type group (Table 1).^19^, ^20^

**TABLE 1:** Classification of families

Family number	No. of carriers	Familial phenotype	*COL3A1* variant (NM_000090.4)	Predicted consequence	Variant type	ACMG Classification
1	1	Severe	Complete deletion	—	Null	Pathogenic (PVS1, PM2, PP3, PP4)
2	4	Unclassified	c.202dupG	p.Asp68Glyfs*33	Null	Pathogenic (PVS1, PM2, PP1)
3	4	Unclassified	c.413delC	p.Pro138Leufs*27	Null	Pathogenic (PVS1, PM2, PP1)
4	1	Unclassified	c.636 + 4A > T	Reduced strength of splice donor site at exon 7 (MaxEntScan score 9.1 > 5.0)	Splice site	Pathogenic (PS3, PM2, PP4, PP5)
5	3	Severe	c.970G > A	p.Gly324Ser	Missense	Pathogenic (PM1, PM2, PP1, PP2, PP3, PP4)
6	13	Attenuated	c.989G > A	p.Gly330Asp	Missense	Pathogenic (PM1, PM2, PP1, PP2, PP3)
7	1	Unclassified	c.1223G > A	p.Gly408Glu	Missense	Pathogenic (PM1, PM2, PM5, PP2, PP3, PP5)
8	3	Attenuated	c.1455 + 5G > A	Reduced strength of splice donor site at exon 20 (MaxEntScan score 9.2 > 3.8)	Splice site	Pathogenic (PS3, PM2, PM4, PP1, PP3)
9	2	Unclassified	c.1573G > A	p.Gly525Ser	Missense	Likely Pathogenic (PM1, PM2, PP1, PP2, PP3)
10	1	Unclassified	c.1662 + 5G > A	Reduced strength of splice donor site at exon 23 (MaxEntScan score 5.5 > −)	Splice site	Pathogenic (PS2, PS3, PM2, PM4, PM6)
11	10	Attenuated	c.1691G > A	p.Gly564Asp	Missense	Pathogenic (PM1, PM2, PM5, PP1, PP2, PP3)
12	1	Severe	c.1923 + 1G > A	Reduced strength of splice donor site at exon 27 (MaxEntScan score 7.6 > −)	Splice site	Pathogenic (PVS1, PM2, PP5)
13	3	Unclassified	c.2283 + 1G > A	Reduced strength of splice donor site at exon 32 (MaxEntScan score 9.3 > −)	Splice site	Pathogenic (PVS1, PM2, PP5)
14	4	Unclassified	c.2510G > A	p.Gly192Ser	Missense	Pathogenic (PM1, PM2, PP1, PP2, PP3, PP5)
15	5	Unclassified	c.2689G > A	p.Gly897Ser	Missense	Pathogenic (PM1, PM2, PM5, PP1, PP5, PP2, PP3)
16	1	Severe	c.2915G > A	p.Gly972Asp	Missense	Pathogenic (PM1, PM2, PM5, PP5, PP2, PP3)
17	1	Severe	c.3039 + 2 T > G	Reduced strength of splice donor site at exon 41 (MaxEntScan score 7.8 > −)	Splice site	Pathogenic (PVS1, PM2, PP3)
18	13	Attenuated	c.3256‐1G > A	Introduction of novel splice acceptor site 1 bp inside exon 45 (MaxEntScan score 5.0)	Splice site	Pathogenic (PVS1, PM2, PP1, PP5)
19	2	Unclassified	c.3302G > T	p.Gly1101Val	Missense	Pathogenic (PM1, PM2, PM5, PP1, PP2, PP3, PP5)
20	6	Attenuated	c.3325C > T	p.Arg1109*	Null	Pathogenic (PVS1, PM2, PP1, PP5,)
21	1	Severe	c.3418‐2A > C	Reduced strength of splice acceptor site at exon 47 (MaxEntScan score 6.2 > −)	Splice site	Pathogenic (PVS1, PS2, PM2, PM6)
22	1	Severe	c.3418G > A	p.Gly1140Arg Reduced strength of splice acceptor site at exon 47 (MaxEntScan score 6.2 > 4.9)	Missense	Pathogenic (PS3, PM1, PM2, PP3, PM5, PP2)
23	1	Unclassified	c.3490G > T	p.Gly1164Trp	Missense	Pathogenic (PS3, PM1, PM2, PM5, PM6, PP2, PP3, PP5)
24	1	Unclassified	c.3490G > A	p.Gly1164Arg	Missense	Pathogenic (PS1, PM1, PM2, PM5, PP2, PP3, PP5)
25	4	Severe	c.3509G > A	p.Gly1170Asp	Missense	Pathogenic (PM1, PM2, PM5, PP1, PP2, PP3, PP5)

All missense variants were glycine substitutions and located in the triple‐helical domain of the α chain of type III collagen (Figure 2). Five of 13 substitutions were to aspartic acid and three were to serin. No significant difference in the proportion of missense variants between families with severe and attenuated phenotype was observed (*p* = 1.00) (Table 2). As expected, families with attenuated familial phenotypes had significantly more affected relatives overall (40 relatives in five families) compared to families with severe familial phenotypes (five relatives in eight families) (*p* = 0.01).

**TABLE 2:** Distribution of genetic variant type by familial phenotype

Variant Type	All variants	Attenuated	Unclassified	Severe
	Number of families (%)			
Total variants	25	5 (20)	12 (48)	8 (32)
Null	4 (16)	1 (25)	2 (50)	1 (25)
Splice site	8 (32)	2 (25)	3 (38)	3 (38)
Missense	13 (52)	2 (15)	8 (54)	4 (31)

### 3.3 Characteristics of patients

Of the 25 index patients, 11 were ascertained due to vascular pathology, six due to characteristic facial features and skin, four due to family history of vascular events, one due to musculoskeletal complaints, one due to malformation caused by amniotic band, one due to recurrent pneumothorax, and one was an incidental finding. Table 3 summarizes the clinical characteristics of index patients and affected relatives.

**TABLE 3:** Characteristics of patients categorized by familial phenotype

Characteristics	All patients	Attenuated	Severe	Unclassified
	Number of patients (%)			
Total	87	45 (52)	13 (15)	29 (33)
Index patients	25 (29)	5 (11)	8 (61)	12 (41)
Relatives	62 (71)	40 (89)	5 (38)	17 (59)
Females	42 (48)	20 (44)	6 (46)	16 (55)
Index patients	14 (56)	2 (40)	5 (63)	7 (58)
Relatives	28 (45)	18 (45)	1 (20)	9 (53)
Major events	32 (36)	16 (36)	9 (69)	7 (24)
Index patients	16 (64)	3 (60)	8 (100)	5 (42)
Relatives	16 (26)	13 (33)	1 (20)	2 (12)
Characteristic facial features^a^	36 (62)	11 (46)	9 (82)	16 (70)
Index patients	13 (72)	0	6 (86)	7 (78)
Relatives	23 (58)	11 (50)	3 (75)	9 (64)
Thin, translucent skin^a^	21 (35)	3 (12)	9 (82)	9 (38)
Index patients	12 (60)	1 (33)	5 (71)	6 (60)
Relatives	9 (23)	2 (9.1)	4 (100)	3 (21)
Easy bruising^a^	29 (48)	11 (44)	7 (63)	11 (46)
Index patients	13 (65)	2 (67)	4 (57)	7 (70)
Relatives	16 (40)	9 (41)	3 (75)	4 (29)
Clubfoot^a^	7 (11)	1 (3.8)	4 (33)	2 (8)
Index patients	4 (18)	0	2 (29)	2 (18)
Relatives	3 (7.3)	1 (4.5)	2 (40)	0
	Years (range)			
Mean age at ascertainment	41 (0–93)	49 (2–93)	21 (0–55)	37 (0–73)
Index patients	32 (2–70)	43 (31–55)	21 (12–30)	35 (2–70)
Relatives	45 (0–93)	50 (2–93)	22 (0–55)	39 (0–73)
Mean age at the first major event or death^b^	48 (12–93)	58 (38–93)	25 (12–55)	51 (37–68)
Index patients	36 (12–68)	49 (41–54)	22 (12–28)	49 (37–68)
Relatives	58 (18–93)	60 (38–93)	37 (18–55)	58 (52–63)
	Number of exposures (%)			
Total interventions	172	72 (42)	46 (27)	54 (31)
Complications	11 (6.4)	4 (5.6)	2 (4.3)	5 (9.3)
Vascular procedures	24 (14)	11 (15)	7 (15)	6 (11)
Complications	4 (17)	1 (9.1)	2 (29)	1 (17)
Surgery of the abdomen	39 (23)	23 (32)	6 (13)	10 (19)
Complications	4 (10)	2 (8.7)	0	2 (20)
Endoscopies	24 (14)	13 (18)	1 (2.0)	10 (19)
Complications	0	0	0	0
Orthopedic surgery	33 (19)	10 (14)	16 (35)	7 (13)
Complications	1 (4.2)	0	0	1 (14)
NANEE^c^ procedures	26 (15)	6 (8.3)	9 (20)	11 (20)
Complications	0	0	0	0
Other procedures^d^	26 (15)	9 (13)	7 (15)	10 (19)
Complications	2 (7.7)	1 (11)	0	1 (10)

^a^ Percentages given in relation to the number of patients assessed for the attribute.

^b^ If no major events were reported for a deceased subject, the date of death was used in its stead. Living patients with no known major complications were disregarded.

^c^ Surgery and invasive diagnostic procedures of the neck, airways, nose, ears, or eyes.

^d^ Other procedures include cesarean section, male and female sterilization, phimosis surgery, lumbar puncture, bone marrow biopsy et cetera.

Characteristic facial features and easy bruising were all more common in patients with severe compared to attenuated familial phenotype, but the difference was not statistically significant. Clubfoot was seen in 33% of examined individuals with severe familial phenotype and 3.8% with attenuated familial phenotype (*p* = 0.03). Thin translucent skin was seen in 82% of examined individuals with severe familial phenotype and 12% with attenuated familial phenotype (*p* < 0.01, but not corrected for multiple testing and not a pre‐planned analysis).

### 3.4 Major events and deaths

Thirty‐two patients (36%) had major events recorded. Sixteen were aortic dissections (11 type A, three type B, and two of unknown location), five were ruptured aortic aneurysms (two thoracic, one abdominal, and two of unknown location), two were dissection or ruptures of the coronary arteries, three were dissections of other medium‐sized arteries, and six were ruptures of other medium‐sized arteries. Sixteen patients died because of their first major event. Five patients survived their first major event but died later due to unknown or unrelated causes. One patient with an unclassified familial phenotype had a stent inserted in his left common iliac artery due to an aneurysm at age 41 and had his first event at 45 with a rupture of the splenic artery only to die at age 49 following a stent thrombosis. Ten of the patients survived a major event and were alive at the last checkup. One patient experienced a bowel rupture, but because the cause was underlying diverticular disease, this was not regarded as a major event.

Of the 28 deceased six deaths occurred without registered major events: one from a cause not related to vEDS, one from hypertrophic cardiomyopathy, and four from unknown causes.

**FIGURE 1:**
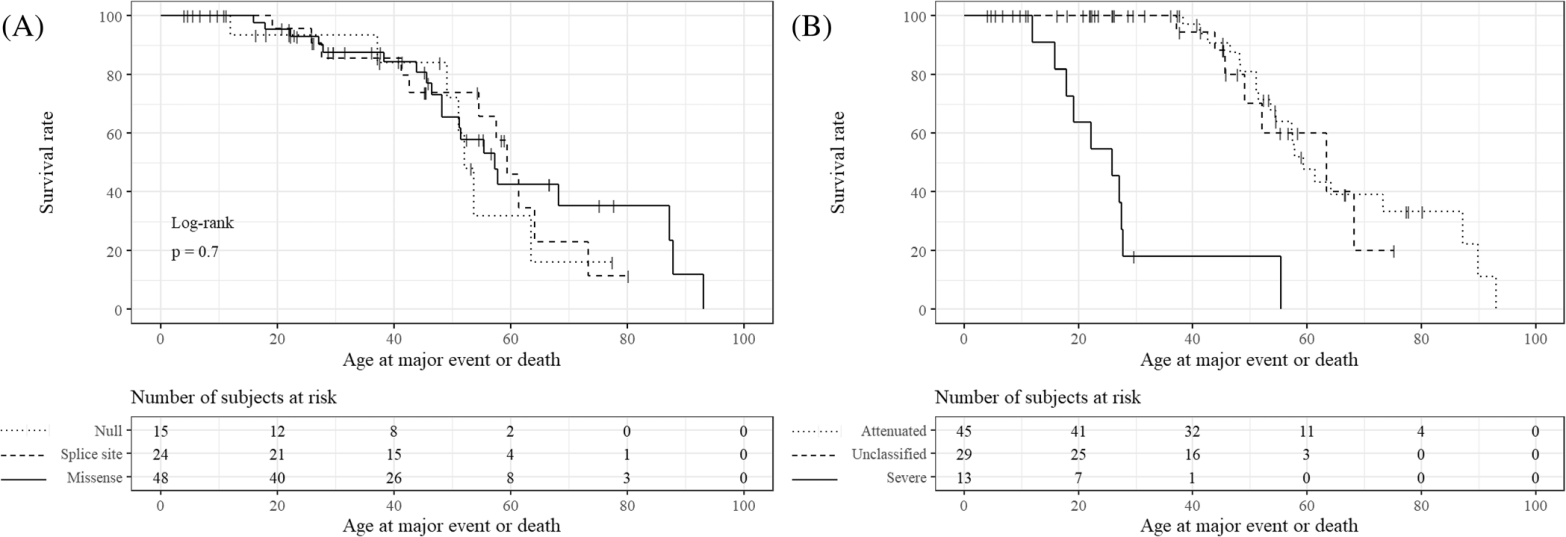
Kaplan–Meier plots comparing the age at the first major event or death. Ticks represent censuring. (A) Patients are categorized according to genetic variant types. The solid line represents missense, the dashed line splice site, and the dotted line null variants. (B) Patients are categorized according to familial phenotype. The solid line represents severe, the dotted line attenuated, and the dashed line unclassified familial phenotype

Age at the first major event or death across variant type and familial phenotype is shown in Figure 1.

**FIGURE 2:**
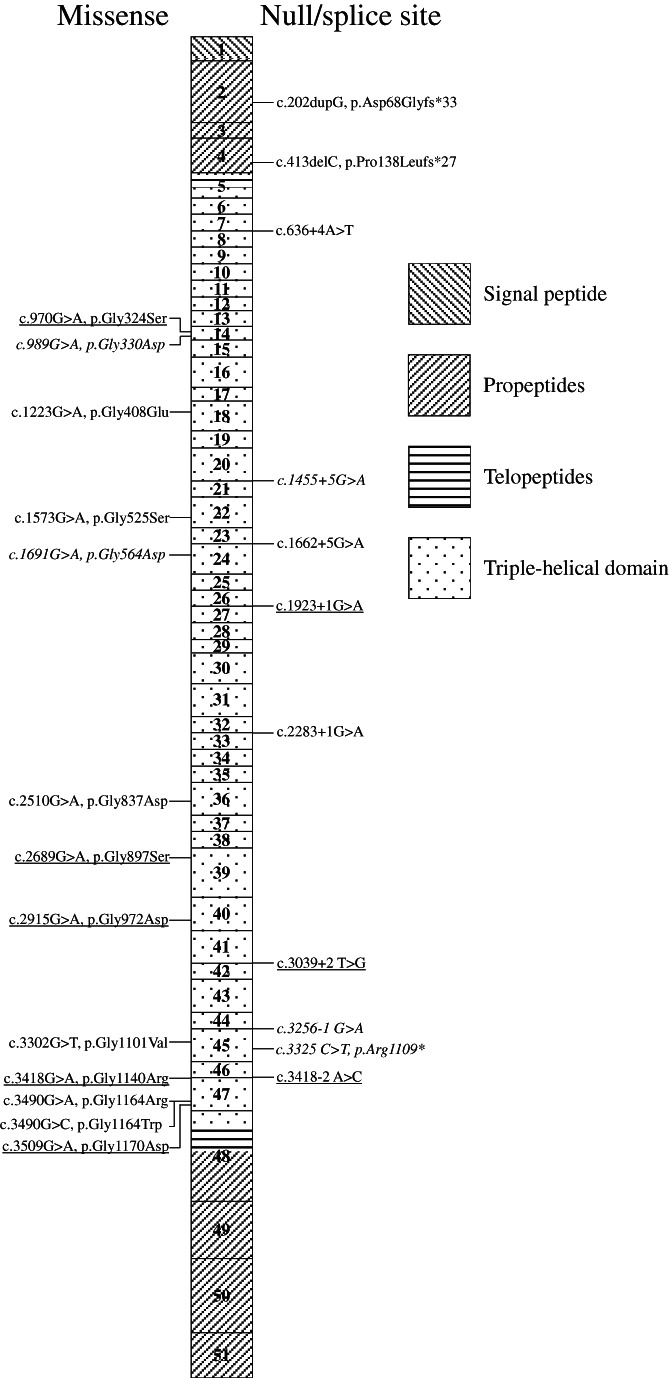
*COL3A1*(NM_000090.3) with variants detected in 25 families with vEDS; one family had a complete deletion (not shown). Boxes represent exons with background pattern denoting protein domains of type III pre‐procollagen.^8^ Variants are coded according to the severity of the familial phenotype: attenuated (italic), severe (underlined), and unclassified (normal)

### 3.5 Deliveries, interventions, and associated complications

Sixty deliveries across 25 women were recorded in the study. Four were registered or recounted as cesarean section. There were no deaths in the peripartum period. Two complications (not counting perineal tears) were recorded, none of these in index patients. One vaginal delivery and one cesarean section were complicated by excessive post‐partum hemorrhaging. In the first case, intrapartum uterine rupture was suspected by the surgeon. Among not‐included at‐risk relatives, one maternal death was reported, namely in the mother of a severely affected patient.

Six index patients underwent 10 deliveries. None of these experienced major complications in their deliveries, although perineal tears were reported in two instances. The six index patients included two from families with attenuated familial phenotype and two from families with a severe familial phenotype. Four carried missense variants and two had splice site variants.

In the 78 medical records available, 172 surgeries or invasive diagnostic procedures in 56 patients were noted, of which 125 were elective. The families with a severe familial phenotype had 46 recorded procedures across 13 patients, whereas families with attenuated familial phenotypes had 72 recorded procedures across 45 individuals. Surgery of clubfoot accounted for 8 out of 16 orthopedic procedures in patients with a severe familial phenotype (Table 3).

Two fatal complications were reported both from acute surgery: One patient with a severe familial phenotype suffered a thrombosis following a carotid cavernous fistula surgery. Another patient from a family with an attenuated phenotype died during reoperation due to post‐operative intraabdominal bleeding after an explorative laparotomy due to acute abdomen with no cause identified. It is unclear whether it was a surgical complication or the original cause of the acute abdomen that necessitated reoperation.

Nine non‐fatal complications to invasive procedures were reported, two of these from families with the attenuated phenotype. The only complication occurring in patients with a null variant was a single case of excessive scar tissue formation after an arthroscopic knee surgery. This was also the only complication to orthopedic surgery.

No complications were recorded as a result of endoscopies and surgical or invasive diagnostic procedures of the neck, airways, nose, ears, and eyes. Table S1 (supplementary) lists all procedures and complications.

## 4 DISCUSSION

This nationwide, retrospective study reports data on all known carriers of pathogenic and likely pathogenic *COL3A1* variants in Denmark.

By identifying patients through all Departments of Clinical Genetics, Centers for Rare Diseases, and specialized units for inherited cardiac conditions in the country, we believe our cohort represents the entire known population of individuals identified with a disease‐causing *COL3A1* variant in Denmark. The 59 living individuals correspond to a prevalence of 1:99000. It is notable that only 29% of patients in this study are index patients, which is far less than other published cohorts.^4^, ^23^

We observed 32 major events in 87 patients. This is much lower than the incidence observed in larger previously published cohorts.^4^, ^10^ Considering the differences in case ascertainment and the distribution of index versus relatives, this is less surprising. The studies by Pepin et al. and Frank et al. both have more index patients than relatives, and a high proportion of index patients ascertained due to vascular events, which enriches the number of events in index patients. In the study by Pepin et al., at‐risk relatives are counted as cases even in the absence of genetic testing, if they have experienced vascular events, enriching the number of events in relatives too. We had access to good pedigree information in 15 of the 25 included families in this study. In these families, nine vascular events possibly attributable to vEDS were reported in first‐degree relatives to known carriers but not genotyped. We have examined how the inclusion of these nine cases would have changed our dataset and find that it does not change our overall conclusions. It does not change classification of familial phenotype either. The event rate would of course have been higher if we selectively allowed persons with events to enter the cohort, but would still be lower than in previous studies. While such an inclusion would make our data easier to compare with other studies, it would also introduce bias. We believe that the lower risk observed and the higher number of ascertained variant‐carrying relatives seen in this study reflects reality in the genomic era and is a consequence of the high completeness and rigor in cascade screening made possible by the Danish health care system and the Danish registries.

Previously, genetic variant type has been used as a tool to subclassify patients based on suggestions that those with null variants in *COL3A1* have a better prognosis.^4^, ^9^, ^10^, ^11^ In line with previous reports^9^, ^10^, ^11^ this study also found that null variants were less common in patients with early severe events. However, predictions based on the variant type and predictions based on our proposed division in familial phenotypes were not always identical. We found two families with vEDS caused by a missense variant with an attenuated familial phenotype. The variants were both substitutions of glycine to aspartic acid in the first half of the collagen triple‐helix domain. Glycine to aspartic acid substitutions in other codons of the domain have both in this study and in previous reports been linked to severe and early events.^4^ Another family with a null variant fell into the severe familial phenotype group, and two families with null variants remained unclassified regarding familial phenotype. Both were small families and could possibly be reclassified as attenuated with more observation time.

It could be that the missense variants we saw in attenuated families are indeed functional null variants. If this is the case, it could be speculated that mRNA sequencing might be useful as a gold standard for severity prediction. If more data regarding familial phenotypes of specific variants are reported in the future, it would be interesting to see if one or more (or a combination of) functional investigations can be used to reliably predict the familial phenotype. Clinically, it is highly desirable to be able to classify phenotype also in de novo cases.

While other groups have reported incidences of bowel perforations of 15–30%, only a single case was observed in the current study.^4^, ^10^, ^24^, ^25^ This could partly be due to the differences in recruitment as discussed previously for Frank et al.^10^ and Pepin et al.^4^, ^25^ Oderich et al.^24^ included only 31 individuals, all of whom had been evaluated in the 30 years up to 2001 and all of whom had at least two major criteria of the 1997 revised Villefranche nosology.^26^ These inclusion criteria are expected to select toward a more severe phenotype. The cohort of Leistritz et al,^9^ including only patients with null variants and thus like our cohort, represents a less severely affected population, reported only vascular complications and no cases of organ rupture. Similarly, while Frank et al.^10^ reported an overall incidence of 30%, the individuals with glycine substitutions had an incidence of 36%, whereas the individuals in less severely affected groups, including null variants, had 0%. It may be that the general disease severity of the cohort irrespectively of the selection method leading to the severity level to some extent predicts the incidence of bowel perforations and that the low incidence in our cohort then in part is explained by the fact that it generally is a less severely affected population.

We developed the familial phenotype classification to explore whether this approach could create an empirical evidence base to allow for more tailored risk evaluations of the patients. A similar subclassification is used for Osteogenesis Imperfecta (OI), a disease comparable to vEDS in that both are collagenopathies, and a milder phenotype has been reported as a result of haploinsufficiency for both.^9^, ^27^ The criteria for subclassification of OI are based on clinical presentation and radiographic findings rather than variant type, and our proposed subclassification follows this example.^28^ Our subclassification of familial phenotype is meant to be used as a supplement not a replacement for the accepted use of variant type in risk assessment of the individual. The proposed set of criteria provides the most weight to major events at a young age in order not to underestimate potential risks. To make the subclassification clinically useful, our proposed criteria allow for a familial phenotype to be classified as attenuated in large families with no major events before the age of 40 and no more than a few major events between the age of 30 and 40, as long as the majority of carriers do not experience any major events until a later age.

Due to the nature of our subclassification, small families are more prone to be classified as having a severe phenotype and large families are more prone to be classified as having an attenuated phenotype. This observation is of course inherent in the nature of the disease where pathogenic variants disposing the patient to early death often will be de novo and simplex cases. Variants leading to a milder phenotype with a lesser effect on reproductive rates will stand a greater chance of providing sufficient observation time to allow for classification by the proposed system. It is a weakness of the classification that a relatively large proportion of patients cannot be classified at the time of diagnosis as they may be the index patient. This choice was made to err on the site of caution and respect the limitations of the available data if the findings are used to implement a less restrictive approach to medical procedures than previously recommended.

In our survival analysis, the proposed familial phenotype classification has higher predictive power than variant type. This cannot be interpreted as proof of validity of the concept because the groups are defined by survival making the observation tautological. Validation in an external cohort, preferably in a prospective study, would strengthen the legitimacy of the classification. It is notable, however, that no major events are observed below the age of 38 in the attenuated familial phenotype, whereas only two patients with the severe familial phenotype attained this age without a major event.

Special precautions regarding pregnancy, including a recommendation for delivery through elective cesarean section, have been advised to patient with vEDS.^14^, ^15^, ^17^ In the large study by Murray et al. examining mortality and morbidity in relation to deliveries, pregnancy‐related deaths occurred in 5.3% of deliveries.^15^ This contrasts markedly with our population, in which not a single pregnancy‐related death was registered. Among not‐included at‐risk relatives, one maternal death was reported, namely in the mother of a severely affected patient. Had we ascertained the population according to the way Murray et al. did, our pregnancy‐related death rate would have been 1 out of 61 (1.6%), proving that the ascertainment procedure is part of the explanation for the divergence in findings. Murray et al.’s approach will overestimate the fraction experiencing events, while our approach will bias the dataset toward patients with successful reproduction. The distribution of index patients versus relatives in Murray et al. is not stated, but in a cohort previously reported by the authors, index patients have outnumbered family members.^15^ As index patients are more likely to have experienced major events, this is also likely to be part of the explanation for the conflicting observations.

Complications to invasive interventions were surprisingly rare. In the entire cohort, no major complications to elective procedures were recorded. While previous recommendations have been restrictive based on a few case reports,^29^ our study suggests that these interventions might be safer than previously thought. As expected, most data regarding elective invasive procedures were obtained from families with attenuated vEDS because this population on average survived to an older age. Very little data were from patients with the severe familial phenotype. Therefore, care must be taken when trying to estimate the safety of invasive procedures for patients from families with the severe familial phenotype based on this study.

Characteristic skin findings and clubfoot appeared to be associated with a more severe phenotype. This is in line with previous observations by Pepin et al.^4^ Based on this finding, we speculate that the minor vEDS criteria may be useful in risk assessment in cases where familial information is too limited for our proposed subclassification to be useful.^30^ Data from the ongoing study of Dutch *COL3A1* variant carriers may refute or confirm this observation.^31^

In clinical practice, it is challenging to strike the right balance between encouraging the patient to live the fullest possible life, while taking the necessary precautions mandated by their disease. Our findings suggest that the rate of pregnancy‐related deaths as well as other major events and surgical complications may be significantly lower in the patients diagnosed in the future. The increased use of extensive genetic testing leads to more families carrying *COL3A1* variants being identified, some of which have very few symptoms of vEDS. As the threshold for genetic analyses is continuously lowered, it is increasingly important to update knowledge regarding penetrance and complication rates to avoid overtreatment and overly restrictive counseling.

## AUTHOR CONTRIBUTIONS

BRD and LR conceived the idea. SS, SF, LR, TBR, and BRD planned the study. SS, RO, SF, DGN, AHC, CRF, PAG, TBR, and BRD contributed to data collection. SS prepared the first draft of the manuscript. All authors contributed to, reviewed, and accepted the final manuscript.

## CONFLICT OF INTEREST

The authors declare no conflicts of interest.

### PEER REVIEW

The peer review history for this article is available at https://publons.com/publon/10.1111/cge.14176.

## Supporting information


Table S1

## Data Availability

Participants of this study did not agree for their data to be shared publicly and due to the nature of this research it is not possible to make supporting data available without compromising anonymity of the participants.
