# Supplementary material for: Carriers of COL3A1 pathogenic variants in Denmark: Interfamilial variability in severity and outcome of elective surgical procedures
Source: Clin Genet. 2022 Jul 4;102(3):191–200. doi: 10.1111/cge.14176 (PMC9544160; doi:10.1111/cge.14176)
Supplement: Supplementary file 1 — Table S1 [file CGE-102-191-s001.docx]

| **Table 4. Recorded surgical and diagnostic interventions and reported complications** | | | | | | | |
| --- | --- | --- | --- | --- | --- | --- | --- |
| ID | Familial phenotype | Variant type | Interventions age <25 | Interventions age 25-35 | Interventions age 35-50 | Interventions age >50 | Complications |
| 1 | Attenuated | Null |  |  |  | Coronary angiography | None |
| 2 | Attenuated | Null | Lumbar puncture  Ear tubes |  |  |  | None |
| 3 | Attenuated | Null |  |  |  | Pacemaker x2  Arthroscopic surgery of the knee | None |
| 4 | Attenuated | Null |  | Gastroscopy | Male sterilization  Surgery for rupture of the Achilles tendon |  | None |
| 5 | Attenuated | Null |  |  | Endoscopic retrograde cholangiopancreatography x3 with one insertion of stent in the bile ducts  Coiling of aneurism of the common hepatic artery blocking the bile ducts  Removal of stent in the bile ducts  Gastroscopy with biopsy  Laparoscopic appendectomy | Elective EVAR of the left common iliac artery with coiling of the left internal iliac artery  Fibrinolysis due to a clot in the stent | None |
| 6 | Attenuated | Splice |  |  |  | Osteosynthesis of fracture of lateral tibial condyle. | None |
| 7 | Attenuated | Splice | Cryptorchidism surgery |  |  |  | None |
| 8 | Attenuated | Splice | Colonoscopy |  |  |  | None |
| 9 | Attenuated | Splice | Inguinal hernia surgery |  |  |  | None |
| 10 | Attenuated | Splice |  |  |  | Acute insertion of stent in the thoracic aorta due to dissection | None |
| 11 | Attenuated | Splice |  |  |  | Cosmetic surgery of the nose  Surgery of scoliosis  Lumpectomy of mammarian cancer | None |
| 12 | Attenuated | Missense | Colonoscopy x2  Surgery of the intestines due to perforated diverticulitis  Clubfoot surgery  Phimosis surgery  Ear tubes | Surgery of the nose due to fractures x2 | Coronary angiography x2  Vascular surgery due to iatrogenic dissection |  | An iatrogenic dissection of the external iliac artery in relation to coronary angiography |
| 13 | Attenuated | Missense |  |  |  | Cataract surgery  Gall stone surgery x3  Proctoscopy with biopsy | None |
| 14 | Attenuated | Missense |  |  | Removal of basal cell carcinoma |  | None |
| 15 | Attenuated | Missense |  |  | Hysterectomy, cause unknown |  | None |
| 16 | Attenuated | Missense |  |  | Surgery for acute abdomen |  | Reoperation due to hemorrhaging, passed away during surgery. Unclear if complication or manifestation of initial cause of acute abdomen |
| 17 | Attenuated | Missense |  | Breast reduction |  |  | None |
| 18 | Attenuated | Missense | Appendectomy |  |  |  | None |
| 19 | Attenuated | Missense | Surgery of bilateral inguinal hernia |  |  | Laparoscopic appendectomy.  Colonoscopy x2, one with polypectomy | None |
| 20 | Attenuated | Missense | Surgery of right inguinal hernia |  |  | Colonoscopy with biopsy  Reoperation of right inguinal hernia x2  Surgery of left inguinal hernia  Unilateral shoulder surgery with decompression, bursectomy and biceps tenetomy | None |
| 21 | Attenuated | Missense |  | Patellar surgery |  |  | None |
| 22 | Attenuated | Missense | Surgical treatment of pyloric stenosis | Caesarean section | Caesarean section  Shoulder surgery due to repeating dislocations  Surgery of hammertoe |  | Second caesarean section resulted in a uterine rupture; a hysterectomy was performed as a result |
| 23 | Attenuated | Missense |  |  |  | Acute resection of the ascending aorta and insertion of a prosthesis due to dissection  Cystectomy and construction of urostomy | None |
| 24 | Attenuated | Missense |  |  |  | Surgery of left inguinal hernia x5  Alloplastic hip surgery  Incision of abscess in the left inguinal region | Fistula with abscess in the left inguinal region, most likely as a result of hernia surgery |
| 25 | Unclassified | Null | Surgery for ileus |  | Elective EVAR due to chronic dissection membrane in the abdominal aorta |  | None |
| 26 | Unclassified | Null | Lingual phrenectomy  Tonsillectomy x2 |  | Arthroscopy of the knee due to lesion of the meniscus  Colonoscopy  Removal of 7 or 8 birthmarks |  | Excessive formation of scar tissue after arthroscopy of the knee |
| 27 | Unclassified | Null |  |  |  | Acute surgery of type A dissecting aneurism of the aorta, insertion of stent. | None |
| 28 | Unclassified | Null |  |  | Coronary angiography and angioplasty |  | Wire-perforation of the left anterior descending artery |
| 29 | Unclassified | Null |  |  |  | Gastroscopy  Colonoscopy | None |
| 30 | Unclassified | Splice | Removal of adenoid vegetations  Ear tube | Gastroscopy  Arthroscopy of the knee due to lesion of the meniscus | Laryngoscopy (videostroboscopy)  Surgery of biceps rupture  Cystoscopy  Surgery of hydrocele  Laryngoscopy with biopsy |  | None |
| 31 | Unclassified | Splice | Exploratory laparotomy  Gastroscopy  Bone marrow biopsy  PEG tube |  |  |  | None |
| 32 | Unclassified | Splice | Left inguinal hernia surgery | Removal of varicose veins |  |  | None |
| 33 | Unclassified | Splice | Removal of salivary gland stone |  |  | Gastroscopy, hiatal hernia  Laparoscopic cholecystectomy due to choledocholithiasis and previous pancreatitis  Hip replacement surgery due to fracture  Bone biopsy | None |
| 34 | Unclassified | Splice | Chest drain x2 due to pneumothorax |  |  |  | None |
| 35 | Unclassified | Missense | Appendectomy |  |  |  | None |
| 36 | Unclassified | Missense | Removal of part of the meniscus | Cholecystectomy with gangrenous and perforated gall bladder  ERCP x2 after the cholecystectomy due to bile leakage | ERCP with insertion of stent in the common bile duct x2 |  | Bile leakage after the cholecystectomy |
| 37 | Unclassified | Missense |  |  | Chest tube insertion |  | None |
| 38 | Unclassified | Missense |  | Hysterectomy due to post-partum hemorrhaging | Removal of ovarian cyst |  | After ovarian cyst post-surgery bleeding, likely due to suture cutting through muscle. Later ileus due to scar tissue formation, treated conservatively |
| 39 | Unclassified | Missense |  |  |  | Percutaneous coronary intervention | None |
| 40 | Unclassified | Missense |  | Caesarean section |  |  | None |
| 41 | Unclassified | Missense | Pyloric stenosis surgery | Cone biopsy of the cervix |  |  | None |
| 42 | Unclassified | Missense | Choanal atresia surgery  Craniofacial reconstruction |  |  |  | None |
| 43 | Unclassified | Missense | Female sterilization  Chevron osteotomy | Gastro-duodenoscopy with biopsies |  |  | The sterilization resulted in a large hematoma at one of the ports. |
| 44 | Unclassified | Missense |  |  |  | Cholecystectomy  Acute stent insertion in left iliac aneurism due to rupture  Coiling of the lienal artery due to rupture | None |
| 45 | Severe | Null | Resection of a 7 cm portion of the descending aorta and insertion of a tube graft due to rupture of the aorta |  |  |  | None |
| 46 | Severe | Splice | Clubfoot surgery x2  Bronchoscopy | Carotid cavernous fistula surgery |  |  | The fistula surgery is complicated with a fatal clot in the medial cerebral artery |
| 47 | Severe | Splice | Balloon valvuloplasty  Tonsillectomy  Chest tube insertion  Colonoscopy |  |  |  | None |
| 48 | Severe | Splice | Cruciate ligament repair | Ligation of renal artery in relation to rupture  Ligation of tibial-fibular trunk  Biopsy from fibroadenoma of the breast |  |  | Subcapsular hematoma of the liver in relation to ligation of renal artery |
| 49 | Severe | Missense | Clubfoot surgery, both feet  Ear tubes  Stenting of the common iliac artery due to dissection  Endovascular surgery due to lesions of the abdominal aorta | Inguinal hernia surgery  Knee surgery after traffic accident |  |  | None |
| 50 | Severe | Missense | Inguinal hernia surgery x2  Clubfoot surgery x3  Ear tubes |  |  |  | None |
| 51 | Severe | Missense | Ear tubes  Removal of neck tumor | Removal of atheroma of the skin  Non-specified orthopedic surgery of the foot |  |  | None |
| 52 | Severe | Missense | Arthroscopic knee surgery x3 | Arthroscopic knee surgery  Caesarean section and sterilization  Chest tube insertion  Lumbar puncture x2  Parathyroidectomy |  |  | None |
| 53 | Severe | Missense | Inguinal hernia surgery  Tenotomy of the Achilles and hamstring tendons |  |  |  | None |
| 54 | Severe | Missense | Clubfoot surgery |  |  |  | None |
| 55 | Severe | Missense | Appendectomy  Laryngoscopy with biopsy |  |  |  | None |
| 56 | Severe | Missense |  |  | Liver biopsy | Bronchoscopy with biopsy | None |
